# Rekrutierung pädiatrischer Praxen für eine Interventionsstudie: Strategie, Umsetzung und Erkenntnisse aus der *Interventionsstudie zur Steigerung der HPV-Impfquoten in Deutschland* *(InveSt* *HPV)*

**DOI:** 10.1007/s00103-026-04256-0

**Published:** 2026-06-09

**Authors:** Johannes Lachmann, Julia Wilhelm, Yvonne Bichel, Jessica Holstein, Heike Kramer, Andrea Mais, Jule M. Schmitz, Selina Teindl, Elisa Wulkotte, Nora Schmid-Küpke, Anja Takla

**Affiliations:** 1 Abteilung für Infektionsepidemiologie, Fachgebiet Impfprävention/STIKO, Robert Koch-Institut, Seestraße 10, 13353 Berlin, Deutschland; 2 Ärztliche Gesellschaft zur Gesundheitsförderung e. V., Hamburg, Deutschland; 3 Institute for Planetary Health Behaviour, Universität Erfurt, Erfurt, Deutschland

**Keywords:** Medizinische Fachangestellte, Pädiatrie, Impfquotensteigerung, Incentivierung, Arztpraxis, Medical assistant, Pediatrics, Vaccination coverage, Incentivization, Medical practice

## Abstract

**Einleitung:**

Die Rekrutierung von medizinischem Personal aus Versorgungseinrichtungen ist in Studien häufig eine Herausforderung, zu der bislang nur begrenzte Evidenz vorliegt. Um diese zu erweitern, berichten wir die im Rahmen der *Interventionsstudie zur Steigerung der HPV-Impfquoten in Deutschland *(InveSt HPV) angewandte Rekrutierungsstrategie und diskutieren diese.

**Methoden:**

Pädiatrische Praxen in Bremen und ausgewählten Landkreisen in Bayern wurden postalisch und über Kanäle des Berufsverbandes der Kinder- und Jugendärzt*innen (BVKJ) kontaktiert. Jede:r Teilnehmende wurde mit bis zu 200 € incentiviert.

**Ergebnisse:**

In Bremen konnten 14 von 39 (35,9 %) und in Bayern 25 von 100 (25,0 %) Praxen für die Studie gewonnen werden, was insgesamt einer Rekrutierungsquote von 28,1 % entspricht. Innerhalb der teilnehmenden Praxen lag die Beteiligung medizinischer und nichtmedizinisch Mitarbeitenden jeweils bei über 90 %. Als Rekrutierungskanäle nannten die Praxen bei Anmeldung am häufigsten die persönliche und schriftliche Ansprache durch den BVKJ, als Teilnahmemotivationen gaben die Praxen insbesondere den Beitrag zur Steigerung der Impfquoten, das Interesse an den Schulungen und monetäre Anreize an. Ein telefonisches Follow-up führte trotz hohen Aufwands kaum zu zusätzlichen Anmeldungen. Für Rückfragen bevorzugten Praxen direkte Kontaktmöglichkeiten gegenüber festgelegten „Sprechstunden“.

**Diskussion:**

Die Erfahrungen aus *InveSt HPV* zeigen, dass die Nutzung bestehender Netzwerke in Kombination mit Incentives in für den Zeitaufwand angemessener Höhe die Rekrutierung von pädiatrischen Praxen begünstigt. Wesentlich erscheinen zudem ein Rekrutierungszeitraum außerhalb der Infektionssaison und Ferienzeiten sowie die frühzeitige Planung ergänzender Maßnahmen für den Fall eines ausbleibenden Rekrutierungserfolgs.

**Zusatzmaterial online:**

Zusätzliche Informationen sind in der Online-Version dieses Artikels (10.1007/s00103-026-04256-0) enthalten.

## Abstract (en)

**Introduction:**

Recruiting healthcare facilities for intervention studies is a well-documented challenge, yet evidence on effective strategies remains limited. To strengthen this evidence base, we report and discuss the recruitment approach applied in the *Intervention Study to Increase HPV Vaccination Coverage in Germany *(InveSt HPV).

**Methods:**

Pediatric practices in Bremen and selected districts in Bavaria were contacted by mail and through the *German Professional Association of Pediatricians* (BVKJ). Each participant received incentives of up to € 200.

**Results:**

In Bremen, 14 of 39 practices (35.9%) and in Bavaria 25 of 100 practices (25.0%) agreed to participate, resulting in an overall recruitment rate of 28.1%. Within participating practices, engagement exceeded 90% among both physicians and non-physician staff. The most frequently reported recruitment channels at registration were personal and written communication via the BVKJ. Reported motivations for participation included contributing to improved vaccination coverage, interest in the training sessions, and financial incentives. Telephone follow-up calls generated very few additional registrations despite considerable effort. For inquiries, practices preferred direct contact options over fixed consultation hours.

**Discussion:**

The experiences from *InveSt HPV* indicate that leveraging existing professional networks in combination with incentives commensurate with the required time investment facilitates the recruitment of pediatric practices. In addition, a recruitment period scheduled outside the infection season and school holidays, as well as the early planning of supplementary measures in case recruitment proves unsuccessful, appear to be essential.

## Einleitung

Eine ausreichende Zahl von Teilnehmenden zu rekrutieren, ist eine Herausforderung für die Durchführung von Studien. Rekrutierungsprozesse werden in wissenschaftlichen Publikationen zwar in der Regel thematisiert, jedoch meist ohne detaillierte Beschreibung der eingesetzten Strategien. Der Großteil der verfügbaren Literatur zu Rekrutierungsstrategien fokussiert dabei auf die Rekrutierung von Patient:innen – zur Rekrutierung von Mitarbeitenden in medizinischen Versorgungseinrichtungen liegt bislang nur eine begrenzte Zahl an Publikationen vor [1, 2].

Eine systematische Übersichtsarbeit von Cochrane zur Rekrutierung für randomisierte Studien im Bereich der Primär- und Sekundärversorgung [1] identifizierte lediglich eine Studie [3], die die Rekrutierung ärztlicher Praxen thematisierte. Darin wurde der Effekt einer postalischen Marketingkampagne evaluiert, die mit einer Kombination aus schriftlicher und telefonischer Kontaktierung und einer Aufwandsentschädigung von 90 € pro Studienpatient:in zu einer Rekrutierungsquote von insgesamt 11,6 % führte. In einer weiteren Publikation wurde die Rekrutierung ärztlicher Praxen über hausärztliche Kollektive der kassenärztlichen Vereinigungen sowie über Lehrpraxen evaluiert [4]. Über mehrere Rekrutierungswellen wurden mit einem mehrstufigen Rekrutierungsplan, bestehend aus postalischem und telefonischem Kontakt, Rekrutierungsquoten zwischen 14 % und 35 % erzielt. Dabei nahmen 5 % der Praxen, die keine Rückantwort auf das initiale Anschreiben gaben, an der Studie teil. Einzelne Interventionsstudien, in denen die angewandten Rekrutierungsstrategien für ärztliche Praxen nicht im Detail beschrieben wurden, zeigen stark variierende Rekrutierungsraten zwischen 2 % und 35 % [5–7]. Ebenfalls zeigen Studien, in denen Netzwerkstrukturen für die Rekrutierung genutzt wurden, eine breite Spannweite der Rekrutierungsraten von 18 % bis 66 %, was unter anderem durch Studiendesign und -dauer beeinflusst wird [8–12]. Unabhängig davon wird in der Literatur wiederholt betont, dass Forschungsnetzwerke einen förderlichen strukturellen Rahmen für Rekrutierungsprozesse bieten: Sie reduzieren den Ressourcenaufwand (Identifikation geeigneter Praxen, Ansprache, Follow-up) und stärken durch kontinuierliche Zusammenarbeit Vertrauen und Bereitschaft zur Teilnahme [8–12]. Zudem scheinen Rekrutierungsstrategien, die auf der Rekrutierung von Ärzt:innen durch ärztliche Kolleg:innen basieren, insbesondere dann erfolgreich zu sein, wenn persönliche Kontakte und bestehende berufliche Beziehungen genutzt werden [13].

Darüber hinaus sind Forschungsnetzwerke in der Primärversorgung in Deutschland bisher nur unzureichend etabliert [4, 14]. An Forschung interessierte Einrichtungen können somit oft nicht gezielt für eine Studienteilnahme kontaktiert werden.

Ziel dieser Originalarbeit ist es, die Evidenz zu Rekrutierungsstrategien für ärztliche Praxen um Erfahrungen aus der *Interventionsstudie zur Steigerung der HPV-Impfquoten in Deutschland (InveSt HPV)* zu ergänzen. Berichtet und diskutiert werden Rekrutierungsstrategie, Rekrutierungsquoten, förderliche und hinderliche Faktoren sowie Herausforderungen.

### Studienkontext: *InveSt HPV*

Vor dem Hintergrund weiterhin niedriger humaner Papillomviren-(HPV-)Impfquoten in Deutschland (2024: bundesweit 55 % der 15-jährigen Mädchen und 36 % der gleichaltrigen Jungen mit einer abgeschlossenen Impfserie [15]) verfolgt die Studie das Ziel, praxisnahe Ansätze zur Förderung der Impfbereitschaft zu untersuchen. Dazu wurde der Effekt bedarfsgerechter Schulungen für Ärzt:innen und medizinische Fachangestellte (MFA) in pädiatrischen Praxen evaluiert. Wir rekrutierten pädiatrische Praxen in den Interventionsregionen Bayern und Bremen und teilten diese zufällig einer von 3 Gruppen zu: Gruppe 1 erhielt eine 2,5-stündige Schulung zu HPV und der HPV-Impfung, Gruppe 2 absolvierte eine 4,5-stündige Schulung zu Gesprächstechniken des *Motivational Interviewing* (MI), während Gruppe 3 keine Schulung erhielt (Kontrollgruppe). Für MFA und Ärzt:innen wurden jeweils angepasste Schulungen entwickelt und angeboten, um den unterschiedlichen Wissensständen und Rollen in der Praxis gerecht zu werden. Die Teilnahme an der Studie beinhaltete für die Interventionsgruppen zudem das Ausfüllen von 10-minütigen Befragungen unmittelbar vor und nach der Schulung sowie ca. 3 Monate nach der Schulung; für die Kontrollgruppe entfiel die zweite Befragung. Aus ethischen Gründen erhielten die Teilnehmenden der Interventionsgruppen nach Abschluss der Interventionsphase und dem anschließenden Beobachtungszeitraum durch ein zweites Schulungsangebot die Gelegenheit, an der bisher nicht absolvierten Schulung teilzunehmen; die Kontrollgruppe konnte eine der beiden angebotenen Schulungen wählen. Die Teilnahme an der zweiten Schulung war keine Voraussetzung zur Studienteilnahme und wurde nicht incentiviert.

## Methoden

### Studienvorgaben für die Rekrutierung

Die Bundesländer Bremen und Bayern wurden als Interventionsregionen ausgewählt, da sie im Jahr 2023 im Vergleich zum Bundesdurchschnitt eine niedrigere HPV-Impfquote aufwiesen. So hatten zu diesem Zeitpunkt 46,5 % der 15-jährigen Mädchen im Bundesland Bremen und 48,9 % der gleichaltrigen Mädchen im Bundesland Bayern eine abgeschlossene Impfserie [16].

Ziel war die Rekrutierung von mindestens 50 pädiatrischen Praxen, darunter mindestens 15 Praxen aus Bremen. Um dieses Rekrutierungsziel zu erreichen, entwickelten wir bundesländerspezifische Strategien. In Bremen ermöglichte die vergleichsweise geringe Zahl niedergelassener Praxen (*n* = 39) einen Kontakt aller 39 pädiatrischen Praxen. In Bayern, dem flächengrößten Bundesland mit über 490 pädiatrischen Praxen, war unter Berücksichtigung der Schulungskapazitäten eine gezielte Beschränkung auf ausgewählte Land- und Stadtkreise erforderlich. Für deren Auswahl wurden folgende Kriterien definiert: (1) Die HPV-Impfquote im jeweiligen Kreis lag unter dem bayerischen Landesdurchschnitt, (2) im jeweiligen Kreis waren mindestens 5 pädiatrische Praxen niedergelassen, (3) die Fahrtzeit zu einem potenziellen Schulungsort in einer nahegelegenen größeren Stadt betrug maximal 45 min.

Auf Basis dieser Kriterien zeigte sich, dass in den Regierungsbezirken Mittelfranken und Niederbayern zu wenige Praxen identifiziert werden konnten, um – unter der Annahme einer realistischen Rekrutierungsquote – ausreichend Teilnehmende für die Durchführung von Schulungen zu gewinnen. Um die Zahl potenziell rekrutierbarer Praxen in diesen Regionen zu erhöhen, wurden in einem weiteren Schritt zusätzliche Kreise innerhalb der jeweiligen Regierungsbezirke einbezogen, die zwar formal nicht die definierte Mindestanzahl niedergelassener Praxen aufwiesen, jedoch sowohl hinsichtlich der Impfquote als auch der Erreichbarkeit eines potenziellen Schulungsortes die Kriterien erfüllten. Durch dieses Vorgehen wurden insgesamt 15 Kreise mit 100 pädiatrischen Praxen identifiziert. Der im Rahmen einer Nachrekrutierung nachträglich einbezogene Stadtkreis München ist hierbei nicht berücksichtigt, da dort aus Zeitgründen – im Gegensatz zu den übrigen Regionen – weder ein postalischer Erstkontakt noch ein strukturierter Aufruf zur Studienteilnahme erfolgte.

Die als Interventionsregionen definierten Landkreise und kreisfreien Städte in Bayern sind in Tab. 1 aufgeführt.

**Tab. 1:** Ausgewählte Interventionsregionen in Bayern mit Anzahl der pädiatrischen Praxen und HPV-Impfquote vollständig geimpfter 15-jähriger Mädchen je Kreis.

Regierungsbezirk	Kreis	HPV-Impfquote (2023)	Anzahl pädiatrischer Praxen
*Oberbayern*	LK Rosenheim	40,4 %	11
	SK Rosenheim	33,6 %	5
	LK Miesbach	41,1 %	5
*Oberpfalz*	SK Regensburg	50,6 %	10
	LK Regensburg	55,2 %	7
*Schwaben*	LK Aichach-Friedberg	43,7 %	6
	SK Augsburg	44,2 %	15
	LK Augsburg	53,7 %	11
*Mittelfranken*	SK Ansbach*	46,5 %	3
	LK Ansbach	46,8 %	6
	LK Weißenburg-Gunzenhausen	47,0 %	5
*Niederbayern*	LK Landshut*	42,7 %	4
	SK Landshut	40,3 %	5
	LK Dingolfing-Landau*	35,4 %	3
	LK Kelheim*	42,3 %	4
*Gesamt*			*100*

Quelle: [15]

*LK* Landkreis, *SK* kreisfreie Stadt

* Nachträglich als Interventionsregion aufgenommen

### Einschlusskriterien

Wir definierten folgende Einschlusskriterien für die Studienteilnahme:

#### Teilnehmendenbezogene Kriterien

- Medizinisches und nichtmedizinisches Personal (z. B. MFA, Gesundheits- und Krankenpflegekräfte), inklusive Personen in Ausbildung
- Volljährigkeit
- Vollständig vorliegende schriftliche Einwilligung zur Studienteilnahme

#### Praxisbezogene Kriterien

- Pädiatrisch tätige Praxis mit Sitz in einer der Interventionsregionen
- Pro Praxis mussten jeweils mindestens 70 % des medizinischen und nichtmedizinischen Personals teilnehmen (z. B. MFA, Gesundheits- und Krankenpflegekräfte), damit ein potenzieller Interventionseffekt auf Praxisebene angenommen werden konnte. Eine „Teilnahme“ ist als eine vollständig vorliegende schriftliche Einwilligung definiert

### Anmeldeprozedere (inklusive Befragung zu Teilnahmemotivation und Rekrutierungskanälen)

Die Anmeldung einer an einer Teilnahme interessierten Praxis erfolgte mehrschrittig, um den Anmeldeprozess besser steuern und administrativ effizienter gestalten zu können.

Im ersten Schritt registrierten sich die Praxen über ein webbasiertes Umfragetool für eine Studienteilnahme. Hierbei wurden die Praxisadresse und Kontaktdaten einer verantwortlichen Ansprechperson für die Studienteilnahme erfasst (siehe Onlinematerial 1: Anmeldung). Eingehende Registrierungen sichteten wir tagesaktuell; neu registrierte Praxen erhielten zeitnah eine Anmeldebestätigung per E‑Mail sowie weiterführende Informationen zum Ablauf der nächsten Schritte.

Im zweiten Schritt baten wir die Praxen, innerhalb von 14 Tagen weitere Angaben über einen zweiten Onlinefragebogen zu übermitteln: (1) Anzahl medizinischer (inklusive Ärzt:innen in Weiterbildung) sowie nichtmedizinischer Mitarbeitenden (z. B. MFA, inklusive Auszubildende), (2) Anzahl der aktiv an der Studie teilnehmenden Mitarbeitenden, (3) bevorzugte Wochentage für Schulungen, (4) Motivation zur Studienteilnahme sowie (5) der Weg, über den die Praxis auf die Studie aufmerksam geworden ist (siehe Onlinematerial 2: Praxisangaben). Die Items zu (4) und (5) wurden durch das Studienteam auf Basis inhaltlich plausibler und kontextbezogener Antwortoptionen entwickelt und wurden nicht vorab getestet; die Angaben hierzu waren optional, eine Mehrfachnennung der gegebenen Antworten war möglich. Bei Überschreitung der 14-tägigen Frist erfolgte zunächst eine Erinnerung per E‑Mail und bei ausbleibender Rückmeldung eine telefonische Kontaktaufnahme.

Im dritten Schritt wurden die schriftlichen Einwilligungserklärungen der Teilnehmenden eingeholt. In den Schritten 2 und 3 wurde geprüft, ob die vordefinierte Mindestteilnahmerate von jeweils 70 % der MFA und Ärzt:innen erreicht wurde. Praxen, die diese Anforderung nicht erfüllten, wurden informiert.

Aufgrund des administrativen Aufwandes und mit Rücksicht auf die Arbeitsbelastung der Praxen zum Zeitpunkt der Rekrutierung während der Infektsaison verzichteten wir auf eine systematische Erhebung von Nichtteilnahme und deren Gründen.

### Incentivierung

Jede teilnehmende Person erhielt eine Incentivierung in Höhe von 200 € (Universalgutscheine). Voraussetzung für die Auszahlung der Incentivierung war die Teilnahme an den Befragungen. Darüber hinaus erhielt jede teilnehmende Praxis ein „Impf-Abenteuer-Kit“, bestehend aus einem Regenmacher, einer Spieluhr, 2 Bilderbüchern und 5 wiederverwendbaren Injektions-Hilfspads (sogenannte Impfigel).

Eine geplante Akkreditierung der Schulung als ärztliche Fortbildungsmaßnahme durch die zuständigen Landesärztekammern konnten wir nicht umsetzen. Ausschlaggebend hierfür war der Einsatz finanzieller Anreize, die im Rahmen von ärztekammerakkreditierten Fortbildungen nicht zulässig sind.

## Ergebnisse

### Rekrutierungsablauf

Anfang Januar 2024 wurden alle pädiatrischen Praxen der Interventionsregionen Bremen und Bayern durch das Studienteam postalisch zur Studienteilnahme eingeladen. Dem Anschreiben waren eine ausführliche Studieninformation, ein Informationsflyer, eine Übersicht potenzieller Vorteile einer Teilnahme sowie eine Anleitung zur Anmeldung beigelegt (siehe Onlinematerial 3 Rekrutierungsmaterialien). Zusätzlich nutzten wir bereits bestehende Netzwerkstrukturen unseres Projektunterstützers, des Berufsverbands der Kinder- und Jugendärzt*innen (BVKJ): Die Landesvorsitzenden in Bremen und Bayern luden Mitte Januar 2024 über interne Kommunikationskanäle sowie durch persönliche Ansprache Praxen aus den Interventionsregionen zur Studienteilnahme ein. Zudem erschien Mitte Februar 2024 ein Artikel im BVKJ-Magazin Kinder- und Jugendärzt*in, der zur Teilnahme aufrief. Des Weiteren nutzten wir Veranstaltungen mit Beteiligung von Ärzt:innen der Interventionsregionen, um die Studie persönlich vorzustellen. Die erste Präsentation erfolgte Ende November 2023 im Rahmen der Jahreshauptversammlung des Landesverbandes Bremen des BVKJ. Interessierte Praxen hatten bereits während der Veranstaltung die Möglichkeit, sich für die Teilnahme anzumelden. Zusätzlich stellten wir die Studie Mitte Februar auf dem Qualitätszirkel Kinder- und Jugendheilkunde Regensburg/Umland Oberpfalz vor und warben dort gezielt für eine Studienteilnahme. Die Nutzung verschiedener Kommunikationskanäle sollte sicherstellen, dass möglichst alle relevanten Praxen über mindestens einen Kanal von der Studie erfuhren. Zur Unterstützung der Rekrutierung implementierten wir eine Webseite mit zentralen Informationen zum Studiendesign, zu den Teilnahmevoraussetzungen, den Vorteilen einer Teilnahme, zum Anmeldezeitraum, einem direkten Link zur Registrierung sowie Kontaktdaten der Studienleitung.

Nachdem wir mehrfach von Praxen um eine Verlängerung der Anmeldefrist gebeten wurden, um sich intern zur Studienteilnahme abstimmen zu können, verlängerten wir den ursprünglich festgelegten Anmeldeschluss um insgesamt 6 Wochen. Dennoch zeichnete sich bereits vor Ablauf der verlängerten Frist ab, dass das angestrebte Rekrutierungsziel in Bayern voraussichtlich nicht erreicht werden würde. Daher ergriffen wir kurzfristig zusätzliche Rekrutierungsmaßnahmen: Zwischen Mitte Februar und Anfang März kontaktierten wir telefonisch alle Praxen in den bayerischen Interventionsregionen, die sich bis dahin noch nicht mit einer Zu- oder Absage zurückgemeldet hatten. Praxen, die innerhalb von 3 Anrufversuchen nicht erreicht wurden oder weitere Informationen wünschten, erhielten eine standardisierte E‑Mail mit den wichtigsten Informationen zur Studie, einem Informationsflyer sowie dem Link zur Onlineanmeldung. Rückfragen konnten per E‑Mail oder telefonisch gestellt werden. Insgesamt wurden 86 Praxen telefonisch kontaktiert, von denen sich 2 Praxen daraufhin zur Studienteilnahme anmeldeten. Von einer vergleichbaren Nachfassaktion in Bremen wurde nach Rücksprache mit der dortigen BVKJ-Vorsitzenden abgesehen. Zusätzlich erweiterten wir Anfang März die Interventionsregionen um den Stadtkreis München. Dank des Aufrufes des dortigen BVKJ-Obmannes konnten 3 weitere Praxen für die Studienteilnahme gewonnen werden, wovon sich eine Praxis nach Rekrutierungsende anmeldete.

Ein Informationsangebot in Form einer „Video-Sprechstunde“, das interessierten Praxen die Möglichkeit bot, Fragen zur Studienteilnahme zu stellen, wurde von keiner Praxis in Anspruch genommen.

**Abb. 1:**
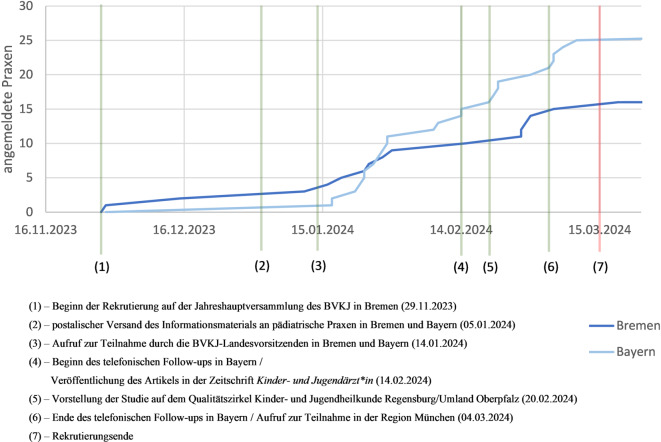
Anmeldezahlen pädiatrischer Praxen in Bremen und Bayern im zeitlichen Verlauf zwischen Rekrutierungsbeginn am 29.11.2023 und dem Rekrutierungsende am 15.03.2024 (*rote Markierung*). *Grüne Markierung*: einzelne Rekrutierungsmaßnahmen. (*BVKJ* Berufsverband der Kinder- und Jugendärzt*innen)

Der zeitliche Verlauf der Anmeldezahlen mit Darstellung der einzelnen Rekrutierungsmaßnahmen ist in Abb. 1 dargestellt.

### Rekrutierungsquoten

In Bremen konnten 14 von insgesamt 39 Praxen rekrutiert werden (35,9 %). Von den in diesen Praxen tätigen Ärzt:innen erklärten 49 von 54 (90,7 %) sowie 105 von 112 nichtmedizinischen Mitarbeitenden (93,8 %) ihre schriftliche Teilnahmebereitschaft. Aus Bremerhaven beteiligte sich keine Praxis.

In Bayern meldeten sich 25 Praxen (25,0 %) mit 76 von 79 Ärzt:innen (96,2 %) sowie 173 von 185 nichtmedizinischen Mitarbeitenden (93,5 %) zur Studienteilnahme an.

Die Rekrutierungsquote lag insgesamt bei 28,1 % der kontaktierten pädiatrischen Praxen (39/139). Innerhalb dieser Praxen erklärten sich 94,0 % der Ärzt:innen (125/133) und 93,6 % der nichtmedizinischen Mitarbeitenden (278/297) zur Teilnahme bereit.

### Befragungsergebnisse zu Rekrutierungskanälen und Teilnahmemotivation

Im Zuge der Anmeldung zur Studie wurde die Ansprechperson stellvertretend für die teilnehmende Praxis gebeten, die Kanäle zu nennen, über die die Praxis auf die Studie bzw. das Schulungsangebot aufmerksam geworden ist. Darüber hinaus wurde die Motivation zur Studienteilnahme erfragt. Mehrfachnennungen waren möglich.

Aus Bremen gingen Angaben von 14 Praxen ein. Die Jahreshauptversammlung des BVKJ wurde von den Praxen am häufigsten als Informationsquelle genannt (*n* = 7), gefolgt von der E‑Mail-Kommunikation (*n* = 4), der persönlichen Ansprache durch BVKJ-Landesvorsitzende (*n* = 3) und der BVKJ-App (*n* = 3).

Aus Bayern erhielten wir Angaben von 25 Praxen. Am häufigsten nannten Praxen die E‑Mail des BVKJ-Landesvorsitzenden (*n* = 9), dessen persönliche Ansprache (*n* = 9) sowie die BVKJ-App (*n* = 5) als Informationswege.

**Abb. 2:**
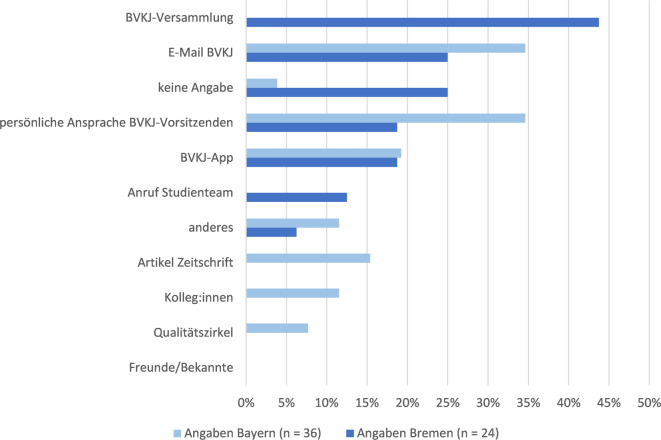
Angaben der teilnehmenden Praxen zur Frage: „Wie sind Sie auf das InveSt HPV-Projekt bzw. das Schulungsangebot aufmerksam geworden?“, Rückmeldungen aus 14 Praxen in Bremen und 25 Praxen in Bayern, Mehrfachnennungen möglich. (*BVKJ* Berufsverband der Kinder- und Jugendärzt*innen)

Der Anteil der einzelnen Rekrutierungskanäle an den Gesamtnennungen, aufgeteilt nach Interventionsregion, ist in Abb. 2 dargestellt.

**Abb. 3:**
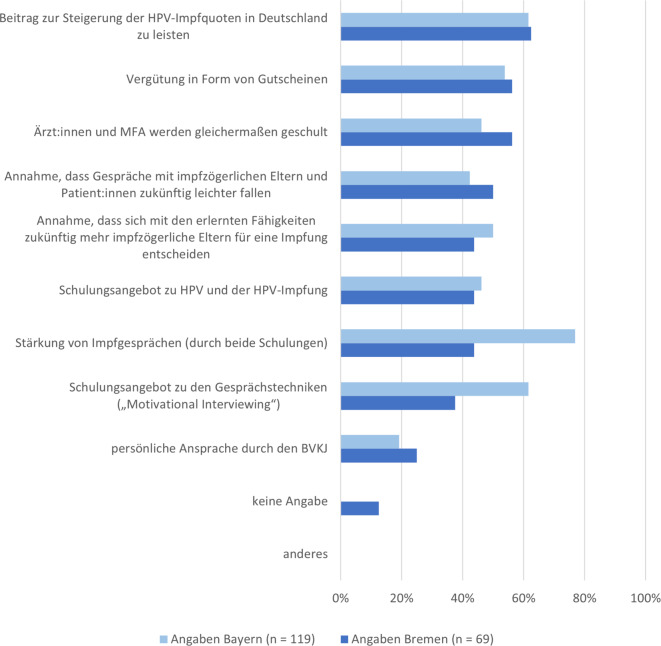
Angaben der teilnehmenden Praxen zur Frage: „Was hat Sie bzw. Ihre Praxis von der Teilnahme an InveSt-HPV bzw. den Schulungen überzeugt?“, Rückmeldungen aus 14 Praxen in Bremen und 25 Praxen in Bayern, Mehrfachnennungen möglich. (*BVKJ* Berufsverband der Kinder- und Jugendärzt*innen)

Als häufigste Teilnahmemotivation gaben die rekrutierten Praxen die Stärkung des Impfgespräches durch beide Schulungen (*n* = 27) und den Wunsch an, einen Beitrag zur Steigerung der HPV-Impfquoten zu leisten (*n* = 26). Darüber hinaus wurden die Aussicht auf Incentives in Form von Gutscheinen (*n* = 23) und die Annahme, dass sich mit den erlernten Fähigkeiten zukünftig mehr impfzögerliche Eltern für eine Impfung entscheiden (*n* = 20), häufig genannt. Auch der Wunsch nach einer Teilnahme an einer der beiden Schulungen wurde oft genannt (MI: *n* = 22; HPV: *n* = 19). Die persönliche Ansprache der BVKJ-Landesvorsitzenden spielte im Vergleich dazu eine untergeordnete Rolle (*n* = 9). In Abb. 3 sind aufgeteilt nach Interventionsregion die Motivationsgründe als Anteil an den Gesamtnennungen dargestellt.

## Diskussion

Im Rahmen von *InveSt HPV* konnten 28,1 % aller pädiatrischen Praxen der Interventionsregionen rekrutiert werden. Die Teilnahmequoten pro Praxis sowohl medizinischer als auch nichtmedizinischer Mitarbeitender lagen jeweils bei über 90 % und damit deutlich über der erforderlichen Teilnahmequote von jeweils 70 % pro Praxis. Das angestrebte Rekrutierungsziel von 50 Praxen wurde mit insgesamt 35 rekrutierten Praxen jedoch nicht erreicht. Aufgrund fehlender Forschungsnetzwerke arbeitete *InveSt HPV* mit dem BVKJ als pädiatrischem Berufsverband. Die auf Praxisebene erzielten Quoten von *InveSt HPV* sind jedoch vergleichbar mit denen von Studien mit Anbindung an Forschungsnetzwerke (zwischen 18 % und 66 % [8–12]).

Die Informationskanäle des BVKJ, etwa eine Veranstaltung des Berufsverbandes sowie die persönliche oder schriftliche Ansprache der Landesvorsitzenden, erwiesen sich als zentral für die Rekrutierung: Ein Großteil der teilnehmenden Praxen gab an, über einen oder mehrere Kanäle des BVKJ auf die Studie aufmerksam geworden zu sein. Diese Beobachtung deckt sich mit den Ergebnissen von Asch et al. [13], die ebenfalls einen förderlichen Effekt bereits bestehender beruflicher Beziehungen auf die Rekrutierung feststellten.

Der postalische Erstkontakt mit umfassenden Studienmaterialien diente zwar der offiziellen Ansprache der Zielgruppe, hatte jedoch aus Sicht der Praxen nur eine geringe Bedeutung für die tatsächliche Rekrutierung. Um Ressourcen zu schonen und den administrativen Aufwand zu minimieren, sollten ausführliche Studieninformationen daher vorzugsweise digital und erst nach einer Registrierung der Praxen bereitgestellt werden. Ein telefonisches Follow-up führte trotz erheblichen Aufwandes nur zu wenigen zusätzlichen Anmeldungen. Diese Beobachtung entspricht den Ergebnissen von Bleidorn et al. [4] und zeigt, dass die telefonische Kontaktaufnahme bei nichtresponsiven Praxen nur begrenztes Potenzial hat. Auch die kurzfristig initiierte Nachrekrutierung im Stadtkreis München führte nur zu wenigen weiteren Anmeldungen. Eine frühzeitige Planung einer Nachrekrutierung wäre aus unserer Sicht sinnvoll gewesen, um bei absehbar unzureichender Rekrutierungsquote schneller und standardisiert zu reagieren, ohne kurzfristige Einzelentscheidungen treffen zu müssen. Konkret umfasst dies: (1) die Definition klarer Entscheidungskriterien für die Auslösung einer Nachrekrutierung, (2) die Vorbereitung einer angemessenen Strategie zur Kontaktierung der Praxen, (3) die Planung der hierfür erforderlichen personellen und zeitlichen Ressourcen, (4) die frühzeitige Einbindung relevanter Multiplikator:innen (z. B. regionale BVKJ-Obleute) sowie (5) die Entwicklung von Mechanismen zum Umgang mit potenziellen Überanmeldungen, um bei hoher Nachfrage eine faire und methodisch konsistente Steuerung zu ermöglichen (z. B. Wartelisten, definierte Priorisierungsregeln). Die Ansprache der Praxen beschränkte sich demgegenüber lediglich auf eine Ad-hoc-Einladung durch den dortigen BVKJ-Obmann. Im Unterschied zu den übrigen Interventionsregionen erfolgte kein postalischer Erstkontakt. Ob ein vergleichbares Einladungsprozedere tatsächlich zu mehr Rekrutierungen geführt hätte, kann jedoch auf Basis der vorliegenden Daten nicht beurteilt werden. Das ergänzende Angebot einer „Video-Sprechstunde“ zur Klärung offener Fragen wurde von den Praxen nicht in Anspruch genommen. Stattdessen suchten sie bei konkretem Informationsbedarf den direkten Kontakt mit der Studienkoordination über E‑Mail oder Telefon. Für zukünftige Rekrutierungsprozesse empfiehlt es sich daher, direkte Kontaktmöglichkeiten für Rückfragen bereitzustellen.

Bei der Rekrutierung von ärztlichen Praxen schien zudem der Rekrutierungszeitraum ein wichtiger Faktor. Dabei sind aus unserer Sicht insbesondere Schulferien sowie gesetzliche Feiertage in den jeweiligen Studienregionen zu berücksichtigen. Darüber hinaus könnten eingeschränkte personelle Kapazitäten und ein erhöhtes Patient:innenaufkommen während der saisonalen Infektionswellen die Rekrutierung erheblich erschweren, was Praxisinhaber:innen während der Rekrutierung bestätigten. Es ist daher anzunehmen, dass der Rekrutierungszeitraum die Teilnahmebereitschaft negativ beeinflusste. Zukünftigen Studien mit kürzerer Interventionsdauer ist aus diesem Grund eine Rekrutierung außerhalb der Infektionssaison zu empfehlen. Das Anmeldeprozedere sollte zudem niederschwellig gestaltet sein, um die Bereitschaft zur Teilnahme zu fördern; insbesondere die Rückmeldung zur Anzahl der teilnehmenden Mitarbeitenden des Praxisteams sollte in einem gesonderten Schritt erfolgen, um den Praxen die Möglichkeit zu bieten, nach einer initialen Praxisanmeldung die Teilnahmen im Team zu organisieren, ohne die Erstanmeldung der Praxis zurückhalten zu müssen. Um diese interne Abstimmung zu erleichtern, sollte der Anmeldezeitraum großzügig bemessen werden. Im Falle von *InveSt HPV* erwies sich ein Zeitraum von 2,5 Monaten zwischen erstem postalischen Kontakt und dem verlängerten Anmeldeschluss als erforderlich.

Die Teilnahme der Praxen war zu einem hohen Anteil intrinsisch motiviert: Am häufigsten wurde der Wunsch, zur Steigerung der Impfquoten beizutragen, sowie die Annahme genannt, dass sich mit den erlernten Fähigkeiten zukünftig mehr impfzögerliche Eltern für eine Impfung entscheiden. Darüber hinaus gaben viele Praxen an, durch die Teilnahme an beiden Schulungen das Impfgespräch stärken zu wollen (Abb. 3). Sozial erwünschtes Antwortverhalten ist hier jedoch nicht auszuschließen. Neben intrinsischen Beweggründen stellte die finanzielle Incentivierung in Höhe von 200 € pro Person einen weiteren relevanten Teilnahmeanreiz dar. Informelle Rückmeldungen von Praxisinhaber:innen deuteten darauf hin, dass insbesondere die Teilnahme des nichtmedizinischen Personals durch die Incentivierung begünstigt wurde. Die Bedeutung der ausgezahlten Incentivierung für die Teilnahmequote wird dadurch gestützt, dass bei identischem Einladungsprozedere zu den Schulungen nach Ende des Interventionszeitraumes, allerdings ohne Incentivierung, nahezu keine Anmeldungen eingingen.

### Limitationen

Als Limitationen sind aufzuführen, dass Gründe für die Nichtteilnahme nicht systematisch abgefragt wurden, sodass wir keine gesicherten Aussagen zu potenziellen Barrieren für die Teilnahme treffen können. Des Weiteren repräsentieren die Angaben zu Informationskanälen und Teilnahmemotivation möglicherweise nur die Perspektive der Ansprechperson, welche die Praxis für die Studienteilnahme angemeldet hat, und unter Umständen nicht das vollständige Meinungsspektrum des Praxisteams.

## Fazit

Die Rekrutierung pädiatrischer Praxen im Rahmen der *Interventionsstudie* *zur Steigerung* *der HPV-Impfquoten in Deutschland* *(InveSt HPV*) verdeutlicht, dass die Nutzung bestehender Netzwerke, in diesem Fall über einen Berufsverband, ein effektiver Ansatz zur Ansprache von Versorgungseinrichtungen sein kann. Eine hohe intrinsische Motivation der Zielgruppe, in Kombination mit einem für den zeitlichen Aufwand angemessenen monetären Anreiz, führte insbesondere vor dem Hintergrund, dass keine forschungsaffinen Praxisnetzwerke einbezogen werden konnten, zu einer vergleichsweise hohen Teilnahmebereitschaft. Ein telefonisches Follow-up zeigte praktisch keinen Effekt. Zusätzliche Maßnahmen bei Nichterreichen des Rekrutierungsziels, wie beispielsweise die Ausweitung der Interventionsregion, sollten frühzeitig geplant werden, um diese bei Bedarf ohne weitere Verzögerungen und strukturiert anwenden zu können. Eine systematische Erfassung der Nichtteilnahmegründe kann helfen, Barrieren zu erkennen und künftige Rekrutierungsstrategien besser zu planen.

Wir erachten es als wesentlich, Rekrutierungsstrategien künftig systematisch und transparent zu berichten. Eine verbesserte Evidenzlage ist unerlässlich, damit zukünftige Projekte von bestehenden Erfahrungen profitieren und die für ihren spezifischen Kontext geeigneten Strategien auswählen können. Darüber hinaus kann die Etablierung gut strukturierter Forschungsnetzwerke dazu beitragen, den Zugang zu Zielgruppen erheblich zu erleichtern und Rekrutierungsprozesse damit zu unterstützen.

## Supplementary Information

Onlinematerial 1: Anmeldung

Onlinematerial 2: Praxisangaben

Onlinematerial 3: Rekrutierungsmaterialien

## Data Availability

Die während der vorliegenden Studie erzeugten und/oder analysierten Datensätze sind auf begründete Anfrage bei der Korrespondenzperson erhältlich.
