# Supplementary material for: Recruitment of pediatric practices for an intervention study: strategies, implementation, and insights from the Intervention Study to Increase HPV Vaccination Coverage in Germany (InveSt HPV)
Source: Bundesgesundheitsblatt Gesundheitsforschung Gesundheitsschutz. 2026 Jun 9;69(7):813–21. [Article in German] doi: 10.1007/s00103-026-04256-0 (PMC13323115; doi:10.1007/s00103-026-04256-0)
Supplement: Supplementary file 1 — Onlinematerial 1: Anmeldung [file 103_2026_4256_MOESM1_ESM.pdf]

InveSt HPV
Interventionsstudie zur Steigerung der HPV-Impfquoten

ROBERT KOCH INSTITUT

Sehr geehrtes Praxisteam,

herzlich willkommen! Auf dieser Seite können Sie sich für InveSt HPV - die Interventionsstudie zur Steigerung der HPV-Impfquoten in Deutschland anmelden.

Nähere Informationen zur Studie entnehmen Sie der Studieninformation oder unserer Studienwebseite unter www.rki.de/invest-hpv-schulungen.

Wir danken Ihnen für Ihre Teilnahme und Ihr Vertrauen in unsere Arbeit!

Mit freundlichen Grüßen,

Die InveSt HPV-Studienleitung
Anja Takla | Nora Schmid-Küpke

Anschrift der Praxis

**Praxisname**	
**Straße und Hausnummer**	
**Postleitzahl**	
**Ort**	

In welchem **Bundesland** befindet sich Ihre Praxis?

Antwort auswählen...

Kontaktdaten der Ansprechperson

Hinweis: Bitte geben Sie nach Möglichkeit nicht die öffentliche Telefonnummer der Praxis an, damit wir Sie bei Rückfragen besser erreichen können.

**Name**	
**Vorname**	
**E-Mailadresse**	
**Telefon**	

optional:
Wenn Sie möchten, können Sie hier Kontaktdaten einer zweiten Ansprechperson angeben.

**Name**	
**Vorname**	
**E-Mailadresse**	
**Telefon**	
