# Supplementary material for: Recruitment of pediatric practices for an intervention study: strategies, implementation, and insights from the Intervention Study to Increase HPV Vaccination Coverage in Germany (InveSt HPV)
Source: Bundesgesundheitsblatt Gesundheitsforschung Gesundheitsschutz. 2026 Jun 9;69(7):813–21. [Article in German] doi: 10.1007/s00103-026-04256-0 (PMC13323115; doi:10.1007/s00103-026-04256-0)
Supplement: Supplementary file 2 — Onlinematerial 2: Praxisangaben [file 103_2026_4256_MOESM2_ESM.pdf]

Angaben zu Ihrer Praxis

Bitte tragen Sie im Folgenden Informationen zu Ihrer Praxis ein.

Anschrift der Praxis

Praxisname	
Straße und Hausnummer	
Postleitzahl	
Ort	

Welche **Praxisform** trifft auf Ihre Praxis zu?

Einzelpraxis

Wie viele **Ärzt:innen** arbeiten in Ihrer Praxis?

Wie viele **medizinische Fachangestellte sowie Gesundheits- und Krankenpfleger:innen** arbeiten insgesamt in Ihrer Praxis?

Angaben zur Anzahl der Teilnehmer:innen

Bitte tragen Sie im Folgenden ein, wieviele Kolleg:innen Ihrer Praxis an der Studie teilnehmen möchten.

Wie viele **Ärzt:innen** werden an der Schulung teilnehmen?

Wie viele **MFAs bzw. Krankenpflegekräfte** werden an der Schulung teilnehmen?

Die Schulungen werden voraussichtlich mittwochs und freitags angeboten.
An welchem Wochentag und zu welcher Tageszeit ist ein Schulungstermin für die teilnehmenden Kolleg:innen am günstigsten?

Mehrfachnennung möglich.

- ☐ Mittwoch vormittags
- ☐ Mittwoch nachmittags
- ☐ Freitag vormittags
- ☐ Freitag nachmittags
- ☐ Anderer Wochentag gewünscht (bitte inklusive Tageszeit):

Wie sind Sie auf das InveSt HPV-Projekt bzw. das Schulungsangebot aufmerksam geworden?

Mehrfachnennung möglich.

- ☐ Persönliche Ansprache durch BVKJ-Landesvorsitzenden
- ☐ E-Mail vom BVKJ
- ☐ Anrufe des Studienteams
- ☐ Artikel in BVKJ Zeitschrift
- ☐ Nachricht in der BVKJ App
- ☐ Auf der BVKJ-Vollversammlung in Bremen
- ☐ Während eines BVKJ-Qualitätszirkels
- ☐ Durch Kolleg:innen anderer Praxen
- ☐ Durch Freunde/Bekannte
- ☐ Anders, und zwar (bitte keine persönlichen Daten über Einzelpersonen nennen):
- ☐ keine Angabe

Was hat Sie bzw. Ihre Praxis von der Teilnahme an InveSt HPV bzw. den Schulungen überzeugt?

Mehrfachnennung möglich.

- ☐ Das Schulungsangebot zu HPV und der HPV-Impfung (ÄGGF-Schulung)
- ☐ Das Schulungsangebot zu den Gesprächstechniken („Motivational Interviewing“)
- ☐ Die Stärkung von Impfgesprächen (durch beide Schulungen)
- ☐ Dass sowohl das ärztliche als auch das nicht-ärztliche Personal zum gleichen Thema geschult wird
- ☐ Die Vergütung in Form von Gutscheinen
- ☐ Die Annahme, dass sich mit den neu erlernten Fähigkeiten zukünftig mehr impfzögerliche Eltern für eine Impfung in unserer Praxis entscheiden
- ☐ Die Annahme, dass Gespräche mit impfzögerlichen Eltern und Patient:innen zukünftig leichter fallen
- ☐ Einen Beitrag zur Steigerung der HPV-Impfquoten in Deutschland zu leisten (durch Erkenntnisgewinn)
- ☐ Die persönliche Ansprache durch den BVKJ
- ☐ Andere, und zwar (bitte keine persönlichen Daten über Einzelpersonen nennen):
- ☐ keine Angabe
