# Supplementary material for: Recruitment of pediatric practices for an intervention study: strategies, implementation, and insights from the Intervention Study to Increase HPV Vaccination Coverage in Germany (InveSt HPV)
Source: Bundesgesundheitsblatt Gesundheitsforschung Gesundheitsschutz. 2026 Jun 9;69(7):813–21. [Article in German] doi: 10.1007/s00103-026-04256-0 (PMC13323115; doi:10.1007/s00103-026-04256-0)
Supplement: Supplementary file 3 — Onlinematerial 3: Rekrutierungsmaterialien [file 103_2026_4256_MOESM3_ESM.pdf]

Teilnahmebedingungen

- Praxen mit pädiatrischer Versorgung im Land Bremen und in ausgewählten Landkreisen Bayerns
- mind. 70% der Ärzt:innen und 70% der MFAs bzw. Krankenpflegekräfte des Praxisteams nehmen teil

Website

Registrierung der Praxis über diesen Link:
www.befragungen.rki.de/InveStHPVAnmeldung

oder über diesen QR-Code

Schulungszeitraum

März 2024 bis voraussichtlich Juni 2024

Jede:r Teilnehmende erhält **Gutscheine** im Gesamtwert von **200 €**.

Zusätzlich erhält jede teilnehmende Praxis ein **Impf-Abenteuer-Kit**.

Kontakt

Wenn Sie Fragen zur Studie haben, schreiben Sie uns gerne eine E-Mail an:

invest-hpv@rki.de

weitere Informationen finden Sie auf unserer Studien-Webseite:

www.rki.de/invest-hpv

Gefördert durch:

Bundesministerium für Gesundheit

aufgrund eines Beschlusses des Deutschen Bundestages

weitere Kooperationspartner und Unterstützer:

UNIVERSITÄT ERFURT

äggf Die ärztliche Gesundheitsbildung

NaLI Nationale Lenkungsgruppe Impfen

Die Senatorin für Gesundheit, Frauen und Verbraucherschutz

Freie Hansestadt Bremen

Herausgeber:
Robert Koch-Institut, Berlin, 2023 | Redaktion: FG33

Das Robert Koch-Institut ist ein Bundesinstitut im Geschäftsbereich des Bundesministeriums für Gesundheit

InveSt HPV

ROBERT KOCH INSTITUT

Interventionsstudie zur Steigerung der HPV-Impfquoten

Einladung zur Studienteilnahme

unterstützt von:

bvkj. Berufsverband der Kinder- und Jugendärzt*innen

Bayerisches Staatsministerium für Gesundheit, Pflege und Prävention

Warum ein Projekt zur Steigerung der HPV-Impfquoten in Deutschland?

Humane Papillomviren (HPV) verursachen Krebs. In Deutschland sind dies fast 8.000 neue Fälle pro Jahr. Die HPV-Impfung bietet einen effektiven Schutz vor diesen Krebserkrankungen.

Derzeit sind lediglich 54% der Mädchen und 27% der Jungen im Alter von 15 Jahren vollständig gegen HPV geimpft – das heißt fast die Hälfte der Mädchen und zwei Drittel der Jungen in Deutschland starten jedes Jahr in ihr junges Erwachsenenleben ohne einen Schutz vor HPV-bedingtem Krebs.

Um der bestehenden Impflücke zu begegnen, führt das RKI die Interventionsstudie zur Steigerung der HPV-Impfquoten in Deutschland (InveSt HPV) durch.

Was wird bei InveSt HPV untersucht?

Ein relevanter Faktor für die Impfentscheidung ist die Arzt-Patienten-Kommunikation. Diese ist vor allem im Gespräch mit unsicheren oder impfkritischen Eltern wichtig. Das Impfgespräch für eine HPV-Impfung wird von medizinischem Personal dabei häufig als besonders herausfordernd eingeschätzt.

Im Rahmen von InveSt HPV werden zwei Schulungsansätze miteinander verglichen, die auf unterschiedlichen Wegen das Impfgespräch stärken. Die geplanten Einmal-Schulungen sind sowohl auf Ärzt:innen wie auch gezielt auf MFAs und Krankenpflegekräfte zugeschnitten.

Was kommt auf uns als Praxis zu?

Nach erfolgreicher Anmeldung wird Ihre Praxis zufällig einer von drei Gruppen zugeteilt:

Gruppe a erhält eine inhaltliche Schulung zu HPV und der HPV-Impfung *(einmaliger Termin, ca. 2 Stunden)*

Gruppe b erhält eine Schulung zu Gesprächstechniken des „Motivational Interviewing“ *(einmaliger Termin, ca. 4 Stunden)*

Gruppe c (Kontrollgruppe) erhält vorerst keine Schulung

Die Teilnahme ist mit einer Befragung am Schulungstermin und einer weiteren Befragung ca. 3 Monate nach der Schulung verbunden (jeweils ca. 10 Minuten). Gruppe c nimmt ohne Schulung an den Befragungen teil.

Allen teilnehmenden Praxen bieten wir im Anschluss die Möglichkeit, an der jeweils anderen Schulung teilzunehmen. Gruppe c erhält ein Teilnahmeangebot für eine oder beide Schulungen.

Welche Fragen werden bei den Befragungen gestellt?

- Allgemeines zur Person (Alter, Geschlecht, beruflicher Hintergrund)
- Einstellungen und Wissen zur HPV-Impfung und den Schulungsinhalten
- Feedback zur Schulung

...und der Datenschutz?

Ihre Daten sind bei uns sicher.

InveSt HPV wurde durch die Datenschutzbeauftragte des RKI geprüft. Ihre Angaben sind pseudonymisiert und werden sicher übermittelt und gespeichert.

Nähere Informationen dazu können Sie der ausführlicheren Studieninformation entnehmen.

Warum sollte sich unsere Praxis an der Studie beteiligen?

- **Höhere Akzeptanz der HPV-Impfung**
  Sie können dazu beitragen, dass mehr Jungen und Mädchen gegen HPV geschützt sind.
- **Zeitersparnis**
  Mit dem richtigen Wissen und den passenden Fähigkeiten wird der Umgang mit unsicheren oder impfskeptischen Eltern leichter.
- **Impfen als Teamaufgabe**
  Wenn das gesamte Praxisteam am Impfprozess beteiligt ist, können Abläufe für alle effizienter gestaltet werden.
- **Schulung als Teamevent**
  Sie können sich mit Kolleg:innen austauschen und lernen gemeinsam Neues. In den Pausen versorgen wir Sie mit leckerem Catering. Die Schulung findet gut erreichbar in Ihrer Nähe statt.

Robert Koch-Institut | Nordufer 20 | 13353 Berlin

Abteilung für Infektionsepidemiologie
FG 33 Impfprävention

Studieninformation zur InveSt HPV-Studie

Versionsdatum:
11.12.2023

Unser Zeichen:
FG33 - Impfprävention

Sehr geehrtes Praxisteam,

vielen Dank, dass Sie sich für die InveSt HPV-Studie interessieren!

Die InveSt HPV-Studie - kurz für „Interventionsstudie zur Steigerung der HPV-Impfquoten in Deutschland" - ist eine wissenschaftliche Studie des Robert Koch-Institutes (RKI).

Im Folgenden erhalten Sie wichtige Informationen zur Studie. Sollten Fragen oder Unklarheiten aufkommen, wenden Sie sich gerne jederzeit an die Studienleitung.

Sie erreichen uns

- per E-Mail unter invest-hpv@rki.de
- postalisch unter der Anschrift:

Robert Koch-Institut
Fachgebiet 33 Impfprävention
InveSt HPV-Studienleitung
Nordufer 20
13353 Berlin.

Mit freundlichen Grüßen

Die InveSt HPV-Studienleitung,

Anja Takla

Nora Schmid-Küpke

Robert Koch-Institut
zentrale@rki.de
Tel.: +49 (0)30 18754-0
Fax: +49 (0)30 18754-2328
www.rki.de

FG33 - Impfprävention
InveSt HPV Studienteam

Anja Takla
Nora Schmid-Küpke

E-Mail:
invest-hpv@rki.de

Besucheranschrift:
Seestraße 10
13353 Berlin

Das Robert Koch-Institut ist ein Bundesinstitut im Geschäftsbereich des Bundesministeriums für Gesundheit.

Hintergrund und Studienziele

Bereits seit 2006 steht ein gut verträglicher und hoch wirksamer Impfstoff zum Schutz vor Humanen Papillomviren (HPV) zur Verfügung. Mit einer zeitgerechten und vollständigen Impfung können die meisten HPV-bedingten Krebserkrankungen verhindert werden.

2021 waren in Deutschland jedoch lediglich 54% der Mädchen und 27% der Jungen im Alter von 15 Jahren vollständig gegen HPV geimpft. Das bedeutet, dass fast die Hälfte der Mädchen und zwei Drittel der Jungen jedes Jahr in ihr junges Erwachsenenleben ohne einen Schutz vor HPV-bedingtem Krebs starten. Die WHO und EU Kommission haben sich das Ziel gesetzt, bis 2030 eine Impfquote von mindestens 90% bei den 15-jährigen Mädchen bzw. eine deutliche Steigerung bei den 15-jährigen Jungen zu erreichen. Dieses Ziel wird auch von Deutschland unterstützt.

Ein relevanter Faktor bei der Impfentscheidung ist das Impfgespräch, um die Sorgen von unsicheren oder impfkritischen Eltern zu adressieren. Medizinisches Personal wird dabei von Patient:innen nach wie vor als vertrauenswürdige Informationsquelle wahrgenommen. Verschiedene Faktoren können die Kommunikation zur HPV-Impfung allerdings erschweren. So bestehen bei einigen Eltern Bedenken gegenüber dem Zeitpunkt der Impfung oder möglichen Nebenwirkungen, andere Eltern wissen nur wenig über die HPV-Impfung und das Virus oder halten die Impfung für nicht notwendig.

Modul 2 der Studie evaluiert daher zwei unterschiedliche Ansätze zur Schulung von Ärzt:innen sowie medizinischen Fachangestellten (MFA) und Krankenpflegekräften und adressiert damit diejenigen Gruppen, die an Gesprächen zur HPV-Impfung am häufigsten beteiligt sind.

Ziel des Studienmodul 2 ist es,

- Schulungen anzubieten, die zielgerichtet auf die Bedarfe des aufklärenden Personals und der Eltern ausgerichtet sind,
- zu eruieren, wie groß der Effekt der jeweiligen Schulungen auf die Impfinanspruchnahme ist
- den praktischen Nutzen der Schulungsangebote im Hinblick auf ein bundesweites Angebot zu prüfen.

Beteiligte Wissenschaftler:innen

Die Studie wird vom Robert Koch-Institut (RKI) durchgeführt. Das RKI ist die zentrale Einrichtung der Bundesregierung auf dem Gebiet der Krankheitsüberwachung und Krankheitsvorsorge.

Die wissenschaftliche Verantwortung für diese Studie liegt bei Anja Takla und Nora Katharina Schmid-Küpke.

Studienablauf

Nach erfolgreicher Anmeldung werden alle teilnehmenden Praxen zufällig einem der drei Interventionsarme zugeordnet. Die Interventionsarme haben folgenden Inhalt:

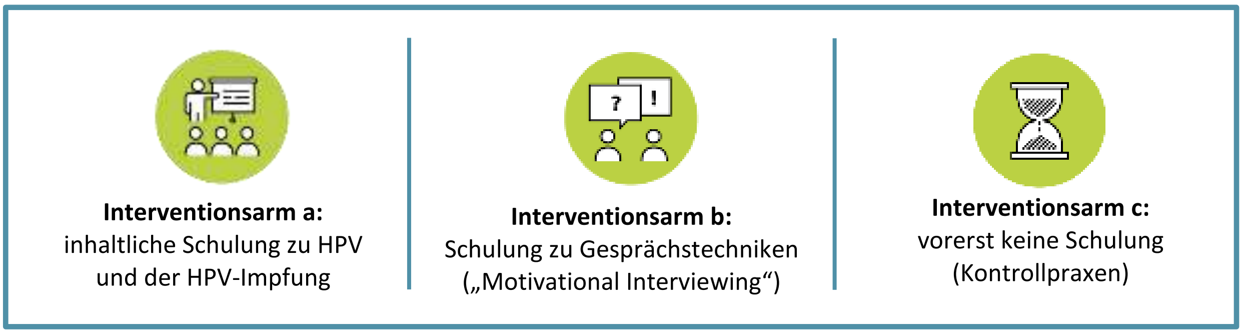

Abbildung 1: Interventionsarme Modul 2 InveSt HPV

Die Schulung im Rahmen des Interventionsarmes erfolgt <u>einmalig</u> im zeitlichen Umfang von ca. zwei (Interventionsarm a) beziehungsweise ca. vier (Interventionsarm b) Stunden. Ärzt:innen bzw. MFAs und Krankenpflegekräfte erhalten die Schulung unabhängig voneinander. Dies ermöglicht es, die Schulungsinhalte besser auf die jeweiligen Berufsgruppen zuschneiden zu können.

Die drei Interventionsarme werden wissenschaftlich begleitet. Dazu füllen die Teilnehmenden zu Beginn und im direkten Anschluss an die Schulung sowie etwa drei Monate nach der Schulung einen ca. 10-minütigen Fragebogen aus. Praxen des Interventionsarms c füllen den Fragebogen im gleichen Zeitraum aus, ohne an einer Schulung teilzunehmen.

Allen teilnehmenden Praxen bieten wir im Anschluss die Möglichkeit, an der jeweils anderen Schulung teilzunehmen. Den Kontrollpraxen werden beide Schulungen angeboten. Somit erhalten bis zum Ende der Studie alle Praxen das gleiche Schulungsangebot.

<u>Geplanter Schulungszeitraum:</u> April-Juli 2024

<u>Schulungsregionen:</u> Land Bremen und ausgewählte* Landkreise in Bayern
(* siehe Tabelle 1 - teilnehmende Kreise Bayerns)

Teilnahmebedingungen

- Pädiatrisch tätige Praxis im Land Bremen oder in ausgewählten* Landkreisen Bayerns (* siehe Tabelle 1 - teilnehmende Kreise des Bundeslandes Bayern)
- ärztliches Personal oder Personen mit Berufsabschluss in einem Gesundheitsberuf (z.B. Medizinische Fachangestellte oder Krankenpflegekräfte)
- mindestens 70% jeder Berufsgruppe (Ärzt:innen sowie MFAs und Krankenpflegekräfte) des Praxisteams erklären sich bereit, an der Studie teilzunehmen Volljährigkeit
- Vorliegende und vollständige Einwilligungserklärung aller Teilnehmenden in der Praxis

Tabelle 1: teilnehmende Kreise des Bundeslandes Bayern

Regierungsbezirk	Kreis
**Oberbayern**	LK Rosenheim
	SK Rosenheim
	LK Miesbach
**Oberpfalz**	SK Regensburg
	LK Regensburg
**Schwaben**	LK Aichach-Friedberg
	SK Augsburg
	LK Augsburg
**Mittelfranken**	SK Ansbach
	LK Ansbach
	LK Weißenburg-Gunzenhausen
**Niederbayern**	LK Landshut
	SK Landshut
	LG Dingolfing-Landau
	LK Kelheim

Aufwandsentschädigung

Für die Studienteilnahme erhält jede:r Teilnehmer:in Gutscheine im Gesamtwert von 200€ des Anbieters „Wunschgutschein“. Dabei wird die Schulungsteilnahme inklusive der Befragung vor und nach der Schulung mit Gutscheinen im Wert von 150€ und die Befragungsteilnahme ca. 3 Monate nach der Schulung mit einem Gutschein in Wert von 50€ bedacht.

Die Teilnehmer:innen der Schulung erhalten darüber hinaus ein Zertifikat über die erfolgreiche Schulungsteilnahme sowie entsprechende Fortbildungspunkte.

Zusätzlich erhält jede teilnehmende Praxis ein „Impf-Abenteuer-Kit“ mit altersgerechten Ablenkungen zur Unterstützung im Impfalltag.

Datenschutz

Das RKI, die Universität Erfurt und die ÄGGF sind für die Verarbeitung personenbezogener Daten im Rahmen der Studie gemeinsam gemäß Art. 26 der Datenschutz-Grundverordnung (DSGVO) verantwortlich. Sie verarbeiten die personenbezogenen Daten nach den Vorschriften der DSGVO und des Bundesdatenschutzgesetzes. InveSt HPV wird durch die Datenschutzbeauftragte des RKI geprüft.

Zum Zweck der Schulungseinladung, Zuordnung zum Interventionsarm, sowie der Vergabe der Gutscheine, des „Impf-Abenteuer-Kits" und der Schulungsevaluation benötigen wir zum Zeitpunkt der Anmeldung folgende Daten:

- Kontaktdaten einer Ansprechperson innerhalb der Praxis (Name, E-Mailadresse, Telefonnummer)
- Anzahl der an der Schulung teilnehmenden medizinischen und nicht-medizinischen Mitarbeitenden (s.o. „Teilnahmebedingungen")
- Kontaktdaten der Praxis (Name, Adresse, Email-Adresse und Telefonnummer)
- Praxisform (Einzelpraxis, Gemeinschaftspraxis/Praxisgemeinschaft, MVZ, andere)
- Sprachen, in denen Gespräche mit Patient:innen geführt werden
- Anzahl der medizinischen und nicht-medizinischen Mitarbeitenden des Praxis-Teams
- Informationsweg (Wie sind Sie auf die Studie aufmerksam geworden?)
- Motivation zur Studienteilnahme
- Nutzung der BVKJ-App in der Praxis

Im Rahmen der Schulungsevaluation (Befragungen) werden folgende Daten erhoben:

- Allgemeines zur Person (Alter, Geschlecht, beruflicher Hintergrund)
- Rückmeldung zur Schulung in Form von Einstellungen und Wissen zur HPV-Impfung und den Schulungsinhalten

Um evaluieren zu können, ob und welche Schulungsmaßnahme den größten Effekt auf eine Steigerung der HPV-Impfquoten hat, erfolgt eine Auswertung der monatlichen HPV-Impfzahlen vor und nach der Schulung nach Interventionsarm. Hierfür werden die Abrechnungsdaten der einzelnen Praxen genutzt, die an die jeweiligen Kassenärztlichen Vereinigungen übermittelt werden. Die Kassenärztliche Vereinigung übermittelt diese anonymisiert und auf Praxisebene aggregiert anhand der praxiseigenen Betriebsstättennummern ans RKI. Um einen möglichen Bias auszuschließen, erfolgt zusätzlich die Übermittlung der vorgenommenen Tdap-Auffrischimpfungen im gleichen Zeitraum.

Die Forschungsergebnisse werden ausschließlich aggregiert und anonymisiert veröffentlicht – dadurch ist auf einzelne Teilnehmer:innen oder die Praxis kein Rückschluss möglich. Die Teilnahme an dem Forschungsprojekt ist freiwillig. Die Erhebungsdaten werden pseudonymisiert auf Servern des Robert Koch-Institutes gespeichert und lassen in aller Regel keine Rückverfolgung auf die Person zu. Eine Identifizierung der Person ist für das Erreichen der Studienziele weder erforderlich noch vom Robert Koch-Institut oder seinen Kooperationspartnern bezweckt. Nach Abschluss der Prozesse werden die praxisbezogenen Daten gelöscht. Die im Rahmen der Schulungsevaluation erhobenen Daten werden im Rahmen guter wissenschaftlicher Praxis zehn Jahre nach Abschluss der Studie gelöscht.

Finanzierung

Die Studie wird aus Mitteln des Bundesministeriums für Gesundheit (BMG) finanziert.

Kooperationspartner

Kooperationspartner der InveSt HPV Studie sind die Universität Erfurt (Philosophische Fakultät, Bereich Gesundheitskommunikation von Frau Prof. Betsch) und die Ärztliche Gesellschaft zur Gesundheitsförderung (ÄGGF) e.V.

Des Weiteren wird das Projekt durch den Bundesverband der Kinder- und Jugendärzte (BVKJ), die Nationale Lenkungsgruppe Impfen (NaLI) sowie das Bayerische Staatsministerium für Gesundheit, Pflege und Prävention und die Senatorin für Gesundheit, Frauen und Verbraucherschutz in Bremen unterstützt.

Kontakt

Ansprechpartner für die Studie ist das Fachgebiet für Impfprävention des Robert Koch-Institutes, vertreten durch

- Anja Takla
- Nora Schmid-Küpke

Sie erreichen uns

- per E-Mail unter invest-hpv@rki.de
- postalisch unter der Anschrift Robert Koch-Institut, Fachgebiet 33 Impfprävention, InveSt HPV-Studienleitung, Nordufer 20, 13353 Berlin.

InveSt HPV Interventionsstudie zur Steigerung der HPV-Impfquoten

ROBERT KOCH INSTITUT

Welche Vorteile haben meine Praxis und ich durch eine Studienteilnahme?

- *Schulung bietet Zeitersparnis im Praxisalltag*
  Die Auseinandersetzung mit impfskeptischen Eltern kann mühsam und zeitintensiv sein. Mit dem richtigen Wissen und den passenden Gesprächstechniken wird der Umgang mit unsicheren oder skeptischen Eltern leichter.

- *Impfen als Teamaufgabe*
  Wenn das gesamte Praxisteam am Impfprozess beteiligt ist, können Abläufe effizienter gestaltet werden. Geschulte Mitarbeitende können erste Gespräche z.B. während der Vorbereitungen für die U- oder J-Untersuchungen mit den Eltern führen, auch wenn sie nicht selbst impfen. Das Aufklärungsgespräch der Ärzt:innen fällt dann unter Umständen kürzer aus.

- *Schulung als Teamevent*
  Die Schulungen finden für Ärzt:innen und nicht-ärztliches Personal getrennt statt und trotzdem: Ärzt:innen und nicht-ärztliche Mitarbeiter:innen aus der Region können sich austauschen und lernen gemeinsam Neues. In den Pausen versorgen wir Sie mit leckerem Catering. Die Schulung findet gut erreichbar in Ihrer Nähe statt.

- Sie erhalten eine Aufwandsentschädigung
  Jede teilnehmende Person aus der Praxis erhält Gutscheine im Wert von 150€ für die Teilnahme an der Schulung sowie einen weiteren Gutschein im Wert von 50€ nach Ausfüllen der jeweils 10-minütigen Befragung

- *Mit jeder Schulungsteilnahme erhalten Sie Fortbildungspunkte*
  Nach erfolgreicher Teilnahme an der Schulung erhalten die Teilnehmenden ein entsprechendes Zertifikat sowie Fortbildungspunkte.

Zusätzlich erhält jede teilnehmende Praxis ein „Impf-Abenteuer-Kit“ mit altersgerechten Ablenkungen zur Unterstützung im Impfalltag.

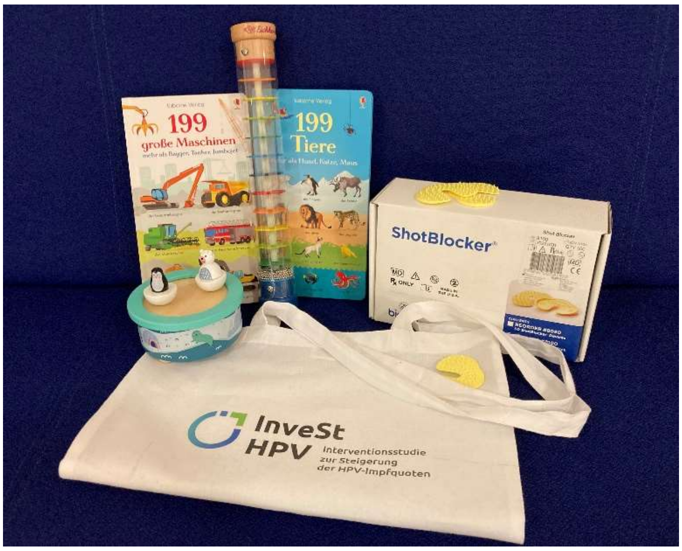

Im Kit enthalten sind:

- ein Regenmacher,
- eine Spieluhr,
- zwei Bilderbücher und
- fünf Impfigel (ShotBlocker®).

InveSt HPV Interventionsstudie zur Steigerung der HPV-Impfquoten

ROBERT KOCH INSTITUT

Anleitung zur Anmeldung für die Teilnahme an der InveSt HPV Studie des RKI

Sehr geehrtes Praxisteam,

bei Interesse zur Teilnahme an InveSt HPV bitten wir Sie, sich bis zum 29.02.2024 online anzumelden.

Schritt 1: Bitte füllen Sie das Online-Anmeldeformular aus.

Auf das Formular gelangen sie über diesen Link
www.befragungen.rki.de/InveStHPVAnmeldung/
oder mit dem nebenstehenden QR-Code.

Folgende Angaben werden dabei benötigt:

- Kontaktdaten der Praxis
- Praxisform
- Anzahl der medizinischen und nicht-medizinischen Mitarbeitenden des Praxis-Teams
- Sprachen, in denen Gespräche geführt werden
- Nutzung der BVKJ-App in der Praxis
- Kontaktdaten einer Ansprechperson innerhalb der Praxis
- Anzahl der Ärzt:innen und Medizinische Fachangestellte bzw. Krankenpflegekräfte, die an der Studie teilnehmen möchten
- bevorzugter Wochentag für den Schulungstermin
- Kanal, über den Sie auf die Studie aufmerksam geworden sind
- Motivation zur Studienteilnahme

Schritt 2: Nach erfolgter Anmeldung (Schritt 1) erhalten Sie eine E-Mail mit dem weiteren Ablauf inklusive eines Formulars zur schriftlichen Einwilligung in die Studienteilnahme. Diese Einwilligungserklärung muss von allen teilnehmenden Kolleg:innen ausgefüllt, unterschrieben und gesammelt per E-Mail an uns zurück geschickt werden. Ohne vorliegende schriftliche Einwilligung ist eine Teilnahme an der Studie nicht möglich.

Nähere Informationen zum Datenschutz finden Sie in der beiliegenden Studieninformation.

Bei Rückfragen zur Anmeldung können Sie sich jederzeit per E-Mail (invest-hpv@rki.de) an uns wenden.
